# How Does the Australian Media Report on the Health and Environmental Impacts of Meat Consumption and Production? A Media Analysis

**DOI:** 10.1002/hpja.70233

**Published:** 2026-08-11

**Authors:** Navid Teimouri, Katherine Sievert, Adam Hannah, Stephanie Meciar, Kerri Gill, Katherine Cullerton

**Affiliations:** ^1^ School of Public Health University of Queensland Herston Queensland Australia; ^2^ Global Centre for Preventive Health and Nutrition, Institute for Health Transformation, School of Health and Social Development Deakin University Geelong Victoria Australia; ^3^ School of Political Science and International Studies University of Queensland St. Lucia Queensland Australia

**Keywords:** environment, framing, meat, media analysis, nutrition, policy

## Abstract

**Issue Addressed:**

Australia is a high meat‐producing and consuming country, yet little Australia‐specific research has examined how news media frame meat's health and environmental impacts and related solutions.

**Methods:**

We analysed 147 Australian print and online news articles published between 15 May 2020 and 15 May 2025 across 12 major outlets. Articles were coded for topic, headline and body framing, calls to action and cited sources, then examined using content analysis and descriptive and inferential statistics.

**Results:**

Coverage focused mainly on environmental impacts (98/147), especially greenhouse gas emissions, while health‐only coverage was less common (33/147). Most articles included a call to action, most often focused on industry action (56) or technological solutions (53), whereas policy change and individual choice were rare (14 each). Industry representatives were the most common sources (91/147) and their inclusion predicted more favourable framing, more technological and industry‐led responses and lower odds of dietary‐change calls. Framing also varied by outlet, with *The Australian* and *Daily Telegraph* more favourable towards meat and the *Sydney Morning Herald* more unfavourable.

**Conclusions:**

Australian news coverage tended to privilege incremental, supply‐side responses over broader dietary and policy approaches recommended in public health and sustainable food systems research.

**So What?:**

Australian news coverage may normalise industry‐friendly and technology‐led responses to meat‐related harms while marginalising dietary and policy solutions, narrowing public and policy understanding of what effective health promotion requires. Strategic media engagement may help health promoters increase the visibility of evidence‐based, structural solutions and support a more balanced public debate about meat‐related harms. Greater government focus on meat‐related harms could help advance public health objectives and policy action.

## 1 Introduction

Australians consume an average of 112 kg of meat per person per year, ranking third globally [1]. Recently, the consumption of meat has become a focal point of public debate [2], driven in part by growing awareness surrounding the environmental impacts of large‐scale meat production and by growing health concerns associated with diets high in red and processed meats (RPMs). While meat can contribute important nutritional benefits and holds cultural importance in many societies [3], excessive consumption (especially RPMs) has been linked with several adverse health outcomes such as obesity, cancer, Type 2 diabetes and cardiovascular disease [3]. Beyond these diet‐related risks, meat production and consumption are also associated with broader public health concerns, including food‐borne and zoonotic diseases [4, 5] and the development of antimicrobial resistance [6].

Meat production also contributes to climate change, with the global livestock sector responsible for 12%–20% of greenhouse gas emissions [7, 8]. Further environmental impacts include land and water scarcity [9], biodiversity loss [10] and nitrogen pollution [11], not to mention a range of animal welfare violations [12]. As early as 2014, livestock was referred to as ‘climate change's forgotten sector’ [13]. Nonetheless, policy attention to reduce the health and environmental harms associated with meat consumption and production has remained limited, particularly, relative to the regulatory focus applied to other major polluting sectors such as electricity and transport [14]. Moreover, policies aimed at reducing meat consumption face low public support [15]. In this context, understanding how these issues are publicly represented becomes critical.

News media is an important contributor to policy and public perception. At the first level, news media plays an agenda‐setting role, meaning that news coverage is an influential factor determining which topics gain attention from the public and governments [16]. At the second level, news media shapes understanding of these issues by highlighting particular details, characteristics and interpretations of the chosen topics [17]. Its coverage does not simply reflect evidence; it frames who is responsible for problems and its solutions, identifies preferred responses and amplifies some voices over others, which can influence how problems are understood by the public and policymakers [18, 19]. In the context of meat specifically, research on animal agriculture and climate reporting has found that media coverage influences how audiences interpret the causes of harm, the actors responsible, and the solutions considered appropriate [20]. Such coverage often emphasises individual, consumer‐oriented responses rather than broader structural changes [21]. However, this framing may vary depending on the ideological slant of the media outlets and their ownership structures behind them [22]. Even and colleagues [23] argue that news media can function as a commercial determinant of health, through agenda setting, framing, source selection and through being used by other commercial actors to influence public and policy debates.

Research on media reporting of meat and its health and environmental implications has so far focused on global, European, United States, Australian and New Zealand Aotearoa contexts [20, 21, 24, 25, 26, 27, 28]. In Australia, media analyses have examined reporting on broad nutritional and environmental issues [18, 29]. However, there has been limited Australia‐specific research examining how news media report on meat consumption and production and its impacts on environmental and human health. The only published Australian study in this space was conducted more than a decade ago and examined the types of meat‐related stories covered in the news and the values that made them ‘newsworthy’ [28]. The lack of more recent analysis is an important gap, particularly given Australia is a high meat producing and consuming country and thus an important population to focus on [1]. Hence, this study will examine how different Australian news outlets report on the nexus between meat and public health. Specifically, it will analyse how the issue is framed, what solutions are proposed, which sources are used, and how these elements interact with each other.

## 2 Methods

We undertook a media analysis of Australian news outlet coverage of meat consumption and production and their impacts on health and the environment.

### 2.1 Data Collection

We searched the Factiva [30] database for online and print articles covering meat consumption or production and their impacts on health or the environment between 15 May 2020 and 15 May 2025. Twelve Australian news outlets were selected to ensure inclusion of the largest national outlets, as well as key state‐based news outlets. Both print and online versions were included if available on Factiva. The search strategy was developed with the support of a public health librarian. The search string combined relevant terms associated with meat (e.g., beef), health outcomes (e.g., obesity) and/or environmental impacts (e.g., greenhouse gas emissions). The complete search string can be found in the Supporting Information.

Articles were exported from Factiva through Endnote [31] into Covidence [32], where duplicates were removed, and all screening was conducted. One reviewer screened all articles, while two other reviewers double screened a total of 20% of articles to ensure rigour. Inclusion and exclusion criteria are described in Table 1.

**TABLE 1:** Calls to action.

Category	Description
Dietary change	The article calls for a change in meat consumption (e.g., people should follow dietary guidelines and limit meat consumption to about 300 g per week).
Policy change	The article calls for policy change regarding meat production or consumption (e.g., the implementation of a meat tax).
Business as usual	The article suggests no changes are required (e.g., describing the status quo as the best possible situation).
Technological solution	The article calls for technological development as a key solution for problems associated with meat consumption or production (e.g., a reduction of on‐farm emissions through feed additives).
Individual choice	The article emphasises the individual choice of each consumer to address problems associated with meat consumption or production through their individual behaviour (e.g., if individuals do not consider this issue important, then no action is needed).
Industry action	The article calls for industry to voluntarily tackle the problems (e.g., industry setting themselves greenhouse gas emissions targets).
Other	Any other CTAs.

### 2.2 Data Analysis

All data was extracted into an extraction template in Covidence. Data extraction included: article title, news outlet, author/s, date of publication, article type, primary topic, secondary topic, framing of headline, framing of article body, mention of scientific studies, call to action (CTA), sources used.

CTAs were classified into categories explained in Table 1. The CTAs may have been explicit or implicit in the article body. Multiple CTAs may have been coded for an individual article.

Headline and article body framing were classified using content analysis and rating each article on a 5‐point ordinal scale reflecting its evaluative stance towards meat production and consumption (1 = *unfavourable* to 5 = *favourable*). For headline ratings, reviewers were given the additional option to choose ‘not applicable’ if the headline did not refer to meat. The article body rating was determined by identifying whether the article focused more on the risks or the benefits of meat consumption or production respectively. If an article predominantly or exclusively stated risks, it was classified as unfavourable (1). If risks and benefits were described, but overall, more emphasis was put on risks, it was rated as slightly unfavourable (2). The same logic was applied for slightly favourable (4) and favourable (5) rating. An article balancing risks and benefits was rated neutral. An overview of benefits and risks is provided in the Supporting Information. To ensure rigour, particularly, among the subjective classification of the headline/body framing, 20% of articles were independently rated by a second reviewer. Inter‐rater‐reliability scores were acceptable for both outcomes (*κ* = 0.67; *N* = 31 for headlines, *κ* = 0.70; *N* = 31 for article body). Any disagreements identified early in this process were resolved through discussion or by consulting a third reviewer. Clarifications to the rating criteria were then made to support consistent coding.

As part of the content analysis, sources—that is, people, organisations, or literature and media quoted by journalists in each article—were also identified. A source was extracted when there was a direct or indirect quote attributed to them, and the name of a person, association or organisation, or similar entity was directly connected to it. Brief mentions of unspecified sources (such as ‘scientists have found …’) were not identified and extracted as individual sources. Table 2 gives an overview of the source classification.

**TABLE 2:** Source classification and description.

Source	Definition
Industry	
Companies/corporations	Commercial companies operating across the meat supply chain, excluding individual farmers (e.g., Coles, Sea Forest, JBS).
Industry associations	Professional associations across the meat supply chain representing industry (e.g., Meat and Livestock Australia, National Farmers Federation).
Individuals	Individuals working in the meat supply chain (e.g., Individual farmers, butchers).
Academia	
Industry‐affiliated academics/institutions	Academics or academic institutions with previous or current ties to the meat industry (e.g., through funding or research collaborations).
Independent academics/institutions	Academics or academic institutions where no identifiable ties to the meat industry were identified.
NGOs/Pro health/environment advocacy groups	
Environmental group	Advocacy groups with a mandate focusing on environmental issues (e.g., Greenpeace, World Wildlife Fund).
Health group	Advocacy groups with a focus on health issues (e.g., Public Health Association Australia, Cancer Council Australia).
Animal welfare group	Advocacy Groups with a focus on animal welfare issues (e.g., PETA, Four Paws).
Farming group	Groups with a focus on farming issues (e.g., Farmers for Climate Action).
Government institutions (e.g., health department, environmental agencies.)	
Politicians	
Intergovernmental organisations (e.g., World Health Organization, International Panel on Climate Change.)	
Individuals not formally affiliated with organisations (e.g., consumers, influencers)	
Other (Sources not captured by the above categories, e.g., journalists, religious groups)	

*Note:* Source extraction was conducted by a primary reviewer and 20% double coded by secondary reviewers. A detailed breakdown of this classification and the comprehensive data extraction template is attached in the Supporting Information.

### 2.3 Statistical Analysis

All analyses were conducted in IBM SPSS Statistics. We reported descriptive statistics (frequencies/percentages) of the article characteristics, framing and CTA patterns, and source utilisation. A set of inferential statistical tests was used to identify associations. To ensure adequate expected cell counts, news outlet outlets were analysed using a six‐level grouping (*ABC*, *Courier Mail*, *Daily Telegraph*, *Sydney Morning Herald*, *The Australian* and Other [*Herald Sun*, *Hobart Mercury*, *The Age*, *Canberra Times*, *West Australian*]). The ‘other’ group was created because the number of articles from the included news outlets in this group was insufficient for statistical analysis. This ‘big‐6’ variable was our primary news outlet factor. When examining change over time, publication year was grouped into 2020, 2021–2022 and 2023–2025 to improve stability.

CTA analyses used the pre‐coded binary indicators (1 = present). Differences in CTA prevalence by outlet group and year group were tested using *χ* ^2^ tests. Co‐occurrence between CTA indicators was assessed using φ coefficients (2 × 2 associations). We examined associations between source types and CTAs in two steps. First, we cross‐tabulated each binary call‐to‐action variable with binary indicators for industry, academic, government, and NGO/advocacy sources and tested bivariate associations using Pearson's *χ* ^2^. Second, we fitted separate multivariable logistic regression models for each call‐to‐action outcome including all four source types as predictors, reporting odds ratios.

Because of sparsity in the five‐level scale, headline framing was collapsed to three categories (unfavourable, neutral, favourable). Differences in headline and article body framing by outlet (big‐6) were examined using *χ* ^2^ tests. Agreement between headline and article‐body framing (three‐level) was assessed using cross‐tabulation. This association was further examined using ordinal logistic regression. To evaluate whether source use predicted article‐body framing, we fitted an ordinal logistic regression including the four binary source‐type indicators (industry, academia, government institutions, NGO/advocacy) and adjusted for news outlet and primary topic; a sensitivity analysis repeated this model using a collapsed three‐level body framing outcome.

Finally, to examine whether academic affiliation was related to framing, articles citing industry‐affiliated academics were compared descriptively and using categorical tests against articles citing independent academics, with Fisher's exact test used where cell counts were small.

## 3 Results

A total of 147 news articles met the inclusion criteria and were included in the analysis (see Supporting Information). The majority of articles reported on the environmental impacts of meat (*n* = 98), while a lower number of articles reported on health impacts alone (*n* = 33) or on both health and environmental impacts (*n* = 16). All news outlets published at least one article on the topics of interest, with larger‐scale news outlets publishing a greater volume of articles. Publication was relatively evenly distributed over time and the sample contained a range of article types, primarily articles reporting on current events. An overview of article characteristics is provided in Table 3.

**TABLE 3:** Article characteristics.

Characteristic	Categories (*n*)
News outlets	*The Australian* (43) *ABC* (27) *Daily Telegraph* (17) *Sydney Morning Herald* (14) *Courier Mail* (13) *Herald Sun* (9) *Hobart Mercury* (8) *The Age* (8) *Canberra Times* (4) *The West Australian* (4)
Year of publication	2020 (12) 2021 (32) 2022 (32) 2023 (31) 2024 (32) 2025 (8)
Article type	News article (104) Feature/longform (23) Opinion piece (13) Analysis (6) Other (1)
Primary topic	Meat and environment (98) Meat and health (33) Both (16)
Secondary topic	Greenhouse gas emissions (104) Diet and health (48) Industry innovation (44) Policy, regulation, or taxes (34) Other sustainability issues (32) Public controversy (18) Meat alternatives (12) Other (2)

### 3.1 Article Topics

**FIGURE 1:**
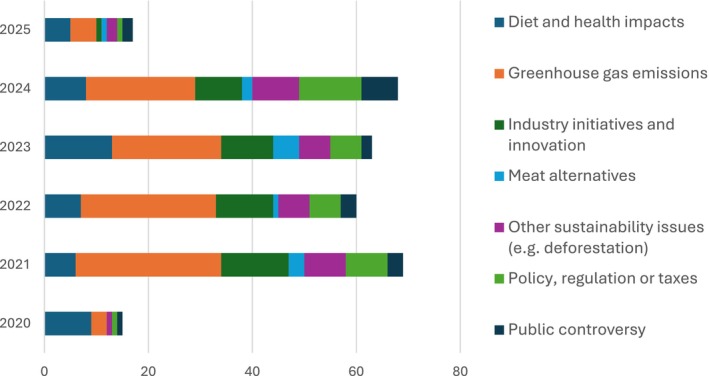
Articles by year and topic. 2020 and 2025 do not include the full year and therefore less articles are included.

There was even distribution of reporting over the included timeframe. Figure 1 visualises the number of articles included the specific topics grouped by year of publication.

Across the sample, environmental topics—particularly greenhouse gas emissions—were the most frequently reported, followed by industry initiatives and policy‐related issues, while health‐focused coverage was less prominent. Within the environmentally focused articles, discussions on methane reduction in animal agriculture were especially prevalent. This included a strong emphasis on feed additives for ruminant livestock as a solution to methane emissions, but also broader political debates on the impact of animal agriculture in contributing to and mitigating methane emissions. In this context, many articles reported on technological developments surrounding the feed additive asparagopsis, an Australian native seaweed with potential to reduce methane emissions in ruminant animals. By contrast, news articles with a health focus often included personal stories involving celebrities or athletes. These stories often reported an individual's health claims without critical assessment of the evidence underpinning them or engaging with relevant academic sources. Instead, the articles focused on the individual experiences of those profiled and presented their claims to the audience with limited contextualisation.

### 3.2 CTA

Of 147 articles analysed, 132 included an explicit or implicit CTA. The most common was ‘industry action’ (*n* = 56) followed by ‘technological solution’ (*n* = 53), ‘dietary change’ (*n* = 39) and ‘business as usual’ (*n* = 26). ‘Individual choice’ (*n* = 14) and ‘policy change’ (*n* = 14) were the least called for actions, and five articles demanded other action. Of the 39 articles calling for a dietary change, the majority (*n* = 34) called for a reduction of meat consumption. A *χ* ^2^ test found a significant association between news outlets and the four most identified CTAs. ‘Business as usual’ (*p* < 0.001) was concentrated in *The Australian* and the *Daily Telegraph* and absent in the *Sydney Morning Herald* and *The Age*. ‘Dietary change’ (*p* = 0.046) appeared relatively more often in the *Sydney Morning Herald* and *Herald Sun*, whereas ‘industry action’ (*p* = 0.004) and ‘technological solution’ (*p* = 0.043) were especially common in the *ABC*, *The Age*, *Hobart Mercury* and *The Australian*. The Supporting Information includes an overview of each newspaper and the percentage of articles that included each CTA.

Interactions between different CTAs revealed several patterns. Technological solution and industry action were often called for together (*φ* = 0.49, *p* < 0.001), while both were negatively associated with dietary change (*φ* = −0.29, *p* < 0.001; *φ* = −0.31, *p* < 0.001). Unsurprisingly, the call for business as usual was negatively associated with all other CTAs. When analysing CTAs over time, we observed little change. The only clear shift was a modest increase in calls for policy change (*χ* ^2^(2) = 8.79, *p* = 0.012) from 2020 to 2023–2025.

CTAs were also tested on their association with the selection of sources included in the article. Articles quoting NGOs or advocacy groups among others had much higher odds of calling for policy change (OR = 4.43, *p* = 0.014). Industry sources were strongly associated with technological solutions and industry action; articles quoting industry sources, alongside other sources, had around six times higher odds of promoting technological fixes (OR = 6.39, *p* < 0.001) and about 14 times higher odds of calling for industry action (OR = 13.64, *p* < 0.001) than articles without industry sources. By contrast, industry sources were much less likely to be linked to a call for dietary change (OR = 0.12, *p* < 0.001), whereas articles citing academics (OR = 2.57, *p* = 0.030) and NGOs/advocacy groups (OR = 4.75, *p* = 0.004) had clearly higher odds of calling for dietary change. No consistent associations emerged between any source type and business‐as‐usual or individual‐choice CTAs. These patterns were consistent with bivariate chi‐square tests based on cross‐tabulated proportions.

### 3.3 Headline and Article Framing

Headline and article body framing were classified based on whether articles emphasised the benefits (favourable) or risks (unfavourable) of meat consumption and production (Table 4).

**TABLE 4:** Framing frequency for headline and article body.

Framing category	Headline, *n* (%)	Body, *n* (%)
Favourable	38 (25.9%)	59 (40.1%)
Slightly favourable	16 (10.9%)	26 (17.7%)
Neutral	42 (28.6%)	21 (14.3%)
Slightly unfavourable	11 (7.5%)	19 (12.9%)
Unfavourable	27 (18.4%)	22 (15.0%)
N/A or unrelated to meat	13 (8.8%)	—

Although article bodies were, on average, more often favourable and less often neutral than headlines, headline and body framing showed substantial alignment at the individual article level. Crosstab analysis showed a high level of consistency between headline and article‐body framing, with 72% exact matches, confirmed by an ordinal logistic regression (OR ≈19.6, *p* < 0.001), indicating that more positive headlines were strongly associated with more positive article bodies. Although *The Australian* had more favourable headlines and the *Sydney Morning Herald* had fewer favourable headlines, these differences were not statistically significant.

Article‐body framing, however, varied significantly by news outlet (*χ* ^2^(10) = 27.6, *p* = 0.002; Cramer's *V* ≈0.29). Specifically, *The Australian*, the *Daily Telegraph*, and the *Hobart Mercury* reported more favourably about the benefits of meat consumption and production, while the *Sydney Morning Herald* reported more on the risks, that is, unfavourably. The *ABC*, *Courier Mail*, and all other outlets occupied an intermediate position. Ordinal logistic regression confirmed these patterns, showing that *The Australian* and the *Daily Telegraph* had the highest odds of reporting favourably (*p* < 0.001). In Table 5, outlets and their frequencies of article framing are displayed.

**TABLE 5:** News outlet and article framing.

News outlet, *n* (%)	Unfavourable	Slightly unfavourable	Neutral	Slightly favourable	Favourable	Total
*ABC*	3 (11)	4 (15)	6 (22)	6 (22)	8 (30)	27
*Canberra Times*	1 (25)	1 (25)	0	2 (50)	0	4
*Courier Mail*	4 (50)	2 (25)	0	2 (25)	0	8
*Daily Telegraph*	2 (12)	0	2 (12)	1 (6)	12 (70)	17
*SMH*	3 (21)	5 (36)	4 (29)	2 (14)	0	14
*Herald Sun*	4 (31)	2 (15)	1 (8)	1 (8)	5 (38)	13
*Hobart Mercury*	0	1 (13)	1 (13)	1 (13)	5 (62)	8
*The Age*	1 (13)	1 (13)	1 (13)	2 (25)	3 (38)	8
*The Australian*	3 (7)	3 (7)	5 (12)	8 (19)	24 (56)	43
*The West Australian*	1 (25)	0	1 (25)	1 (25)	1 (25)	4
Total	22 (15)	19 (13)	21 (14)	26 (18)	59 (40)	147

*Note:* Percentages may not add to 100% due to rounding.

### 3.4 Representation of Sources Cited in News Coverage

Analysis of the sources quoted in the articles indicated that industry representatives were by far the most included sources in the analysed news articles. Ninety‐one articles (62%) included at least one industry source; of these 28 (19% of total) included no other type of source. Fifty‐seven articles included academics (39%), 33 politicians (22%) and 31 NGOs or advocacy groups (21%). Other sources included government institutions (*n* = 26, 18%), individuals from the public (*n* = 18, 12%), health professionals (*n* = 16, 11%) and intergovernmental organisations (*n* = 11, 7%). Twelve articles included at least one source not classifiable in the previous categories. Industry, academic, and NGO sources were further classified into sub‐categories, which are displayed in Table 6.

**TABLE 6:** Sources used with frequencies.

Source group	Subcategory	*N* (% of all articles (*n* = 147))
Industry	Total industry sources	91 (62)
	Corporations/companies	51 (35)
	Industry associations	48 (33)
	Individuals	15 (10)
	Other	1 (1)
Academia	Total academic sources	57 (39)
	Independent academics	29 (20)
	Industry‐affiliated academics	15 (10)
	Both	2 (1)
	Unclear affiliation	11 (7)
NGOs/advocacy groups	Total NGO/advocacy	31 (21)
	Health	14 (9)
	Environmental	14 (9)
	Farming	3 (2)
	Animal‐welfare	3 (2)
	Other	1 (1)

The inclusion of industry sources varied significantly by news outlet (*χ* ^2^(5) = 20.05, *p* = 0.001; Cramér's *V* = 0.37). Industry sources were overrepresented in both the *ABC* and *The Australian*, appearing in 82% and 79% of their articles, respectively. However, only four (15%) *ABC* articles relied exclusively on industry sources compared with 15 articles (35%) in *The Australian*. In contrast, only 31% of *Courier Mail* articles included industry sources and other outlets used them in around 50% of their articles. The use of industry sources also differed strongly by primary topic (*χ* ^2^(2) = 39.26, *p* < 0.001; *V* = 0.52). Industry sources appeared in 79% of environment‐focused articles, compared with only 18% of health‐focused articles and 50% of articles covering both topics. Use of academic sources did not differ significantly across outlets overall.

An ordinal logistic regression adjusting for outlet and primary topic showed that citing industry sources predicted approximately four times higher odds of more favourable article framing (aOR = 3.89, 95% CI = 1.66–9.15, *p* = 0.002). Of the articles that relied exclusively on industry sources, none reported unfavourably about meat. In contrast, citing NGO/advocacy sources was associated with substantially lower odds of more favourable framing (aOR = 0.24, 95% CI = 0.10–0.57, *p* = 0.001). The model also showed that articles reporting on environmental issues had approximately five times higher odds of reporting favourably compared to articles covering both or only health issues (aOR = 5.29, 95% CI = 1.83–15.29, *p* = 0.002). Model fit was adequate (Nagelkerke *R* ^2^ = 0.427; Deviance *p* = 0.988), but some cells were sparse, so results were confirmed in sensitivity analyses using a three‐level outcome.

A closer examination of academic sources showed that affiliation mattered. Articles citing industry‐affiliated academics were far more likely to portray meat favourably (*χ* ^2^(2) = 13.97, *p* < 0.001; Cramér's *V* = 0.55). Specifically, 71% of articles including industry‐affiliated academics had favourable framing towards meat (12/17), compared with 28% citing independent academics (8/29). Favourable framing was about six times more likely when the cited academic was industry‐affiliated (OR ≈6.3).

The choice of included academic sources demonstrated the specificity of topics and associated academics. The academics quoted tended to be highly specialised academics in narrow technical domains, such as seaweed research. Academics with a focus on broader environmental or health impacts of meat production or consumption were not included as frequently.

## 4 Discussion

Our study provides a focused examination of Australian news outlet coverage of meat production and consumption and its impacts on health and environmental outcomes. Across 147 news media articles over 5 years, clear differences in reporting methods and outcomes were identified. There was sustained media interest in meat consumption and production from 2020 through to 2025, with particular emphasis on meat's environmental impacts, which were reported more frequently than health impacts. In contrast, an Australian media analysis from 2014 found that health‐related topics were more prominent than environmental impacts in meat related news coverage [28]. This suggests that environmental reporting in connection with meat has become increasingly prominent over the past decade [28]. This may be due to increased awareness and media reporting of the environmental impacts of the global livestock industry, alongside growing calls for an aggregate reduction of global meat production to achieve global climate targets [26, 33, 34]. Additionally, reporting on meat's environmental impacts may have escalated following the publication of several large scientific reports [35], such as the EAT‐Lancet report ‘Food in the Anthropocene’ or the IPCC Special Report on Climate Change and Land, which were both published in 2019 [33, 36]. All included news outlets had published articles connected to this topic during the five‐year study period, with the *ABC* and *The Australian* publishing the majority of articles. However, previous research indicates that overall media reporting on meat is often dominated by other topics such as animal welfare or the economic impacts [28], limiting the public's exposure to meat's public health impacts.

Overall, coverage was characterised by relatively favourable or neutral framing towards the benefits of meat consumption and production, a reliance on industry sources, and calls for action that emphasised technological solutions and industry‐led responses rather than more structural changes such as dietary shifts or policy and regulatory measures that could reshape food systems. These patterns suggest that Australian news media are, at present, more likely to report on incremental, supply‐side approaches for addressing meat's environmental impacts rather than the more transformative changes recommended by planetary health and sustainable diet frameworks [33].

Most articles included an explicit or implicit CTA. Previous research has found that media reporting often uses the frame of individual choice when referring to the environmental impact of meat consumption, positioning change at the level of the individual, rather than policy, economic or organisational interventions [20, 21, 37]. In our analysis, calls for individual choice were rare, while calls for industry‐based action and technological solutions were common. This may be explained by the high number of articles that relied on industry representatives as their sources, who were 6 and 14 times more likely to call for technological solutions or industry action, respectively. As industry actors tend to oppose government interventions that may constrain their business practices and instead advocate for self‐regulation or innovation‐based solutions, it is unsurprising that our findings align with previous research [38, 39]. The meat industry in particular has consistently resisted policies aimed at reducing meat consumption [24].

Across all 147 articles, industry representatives were the most common source used and were present in 62% of articles. This contrasts with an earlier Australian media analysis of articles reporting on nutrition policy from 2008 to 2018 which found that academics were the most commonly quoted source, while the food industry was only quoted in 21% of articles [18]. When looking at articles reporting on meat and its health impacts specifically, however, academics were the most used source (36% of articles) and industry sources were only used in 17% of articles. This suggests that there are differences in sourcing practices related to the themes investigated, with industry more present in reporting on environmental issues. Nonetheless, the findings confirm previous research which indicates that the meat industry attempts to influence media reporting to their benefit [24].

Unsurprisingly, articles quoting industry sources were less likely to call for dietary change, consistent with the commercial interests of meat industry actors in maintaining consumption levels and protecting profit margins. Previous analysis of Australian media has similarly shown that reporting on food systems and climate change emphasises production‐side impacts rather than linking food consumption with environmental issues [29]. This emphasis sits at odds with increasing scientific evidence that dietary shifts, which are shaped by food environments, market concentration, and broader structural conditions, are necessary to reduce food systems' impacts on environmental degradation [34, 40]. The relative marginalisation of dietary‐change CTAs in Australian news coverage may therefore contribute to the public's persistent underestimation of food's climate impacts and the reluctance to reduce meat consumption documented in previous studies [19]. When Australia committed to methane emissions reductions through the 2023 ‘methane pledge’, the government similarly focused on technological solutions over measures that would actively reshape consumption (such as pricing, procurement, or dietary guidance) [41]. This political framing may explain the strong media focus on reduction technologies.

Compared with technological solutions or industry action, explicit calls for policy change were exceptionally rare in our analysis, and were most frequently voiced in articles including NGOs and advocacy groups as sources. This pattern reflects longstanding observations in behavioural sciences, health, and climate change literature that public discourse often privileges individual and industry‐led change over systemic or regulatory policy change, a tendency linked to detrimental health and environmental outcomes [42, 43, 44, 45]. Moreover, our findings suggest that media debates frequently construct mutually exclusive solution pathways—either technological and industry‐led or demand‐side dietary change—rather than presenting them as complementary levers within broader food systems transformation.

In contrast to previous research from the United Kingdom [25, 26], our analysis revealed a predominantly pro‐meat sentiment orientation in Australian media reporting. Over half of the articles reported favourably towards meat, while only 28% reported unfavourably. This finding can be partly explained by the substantive focus of the sample. Many articles centred on Australian technical solutions to ruminant methane emissions, specifically the development of feed additives including *the Australian* native seaweed Asparagopsis, originating from the government‐industry research institute, CSIRO. Several Australian companies are now commercialising this technology [46, 47], generating large‐scale media attention. The favourable tone accompanying this coverage may reflect active industry communication strategies. Australian research has shown that food industry actors routinely use public relations tactics to generate favourable news coverage of their products [48].

Given that headlines and article content have previously been found to diverge, with headlines often designed to attract attention and acting as macro‐level summaries that can disproportionately influence readers' interpretation of articles [49, 50], we anticipated differences in favourability framing between headlines and article content. However, our analysis found strong alignment between headlines and article framing. Article body framing and CTA were significantly influenced by the publishing outlet. NewsCorp owned publications, *The Australian*, the *Daily Telegraph*, and the *Hobart Mercury*, were more likely to report favourably about meat and include a ‘business as usual’ CTA, while the *Sydney Morning Herald* was more likely to report unfavourably and support the ‘dietary change’ CTA. This pattern aligns with research conducted on Australian news media reporting on environmental coverage [22]. NewsCorp outlets repeatedly were identified as drivers of climate scepticism and have a documented history of casting doubt upon topics of scientific consensus and supporting industry‐friendly narratives [51]. Further research could explore these findings through a political economy lens examining the influence of economic power on public discourse [52].

Our findings have important implications. The prominence of industry sources alongside limited representation of policy‐oriented change CTAs suggests that Australian news coverage may be conducive to voluntary, supply‐side, and technological solutions, supportive of an agenda encouraging little change in demand‐side transformation. However, evidence on sustainable diets clearly indicates that these solutions alone are unlikely to achieve health and environmental targets without broader shifts across food systems, including a reduction in meat consumption [53], particularly in high‐income countries like Australia [33, 54]. Although there is a variety of options on how to achieve sustainability targets [55], public health and environmental advocates may need to engage more strategically with media outlets using specific news values to ensure dietary and policy solutions are represented as legitimate and effective solutions [56]. The strong media focus on industry and technological solutions can lead to unrealistic expectations by the public that sustainability and health aims can be achieved without changes to consumption patterns. For policymakers, this may create challenges if such innovations prove insufficient and demand‐side policies become necessary.

This study has several strengths, including its detailed focus on Australian news coverage of meat across both health and environmental domains, its inclusion of major outlets covering all federal states over a five‐year period, and its systematic coding of framing, CTAs and source use. However, the study also has limitations. Some analyses were based on small cell counts, requiring category collapse and cautious interpretation. Framing assessments also involved an unavoidable degree of subjective judgement; however, double coding was undertaken to help mitigate this. Our study describes patterns in coverage but cannot establish their effects on public opinion or policymaking. Finally, while we identified that sources have an important role in shaping media reporting, the mechanisms behind this remain unclear. Future research may investigate how different sources impact reporting, for example, by analysing the use of interest group press releases in media articles.

## 5 Conclusion

Our study examined how major Australian news outlets report on meat consumption and production in relation to health and environmental outcomes. We found that coverage was shaped not only by the topics selected for reporting but also by the framing of meat, sources used and types of solutions called for. Overall, Australian news coverage tended to be favourable towards meat products and companies, relied predominantly on industry sources for information, and focused on technological and industry‐led solutions to tackle the public health harms associated with the current levels of meat consumption and production. Given the important role of news media in shaping public perceptions and policy implications, these findings highlight the need for public health advocates to engage with news outlets to ensure balanced and evidence‐based reporting to the public. Increased attention from public health organisations and governments to meat‐related harms and impacts may in turn generate additional and potentially more balanced and evidence‐based news coverage on these issues.

## Author Contributions

N.T., K.S., A.H. and K.C. developed the research question and objectives. Screening and data extraction were conducted by N.T., K.G. and S.M. Data analysis was done by N.T. with oversight from K.C. The final manuscript was written by N.T. with substantial support from K.S., A.H. and K.C. All authors made substantive intellectual contributions to all aspects of the research and writing of this article and approved the final version.

## Funding

The authors have nothing to report. N.T., K.G. and S.M. are recipients of an Australian Government Research Training Program Scholarship for higher degree by research students.

## Ethics Statement

The authors have nothing to report.

## Conflicts of Interest

The authors declare no conflicts of interest.

## Supporting information


**Table S1:** Inclusion and exclusion criteria.
**Table S2:** Benefits and risks of meat production/consumption.
**Figure S1:** PRISMA flowchart.

## Data Availability

The data that support the findings of this study are available from the corresponding author upon reasonable request.
