# Supplementary material for: How Does the Australian Media Report on the Health and Environmental Impacts of Meat Consumption and Production? A Media Analysis
Source: Health Promot J Austr. 2026 Aug 11;37(4):e70233. doi: 10.1002/hpja.70233 (PMC13459751; doi:10.1002/hpja.70233)
Supplement: Supplementary file 1 [file HPJA-37-0-s001.docx]

## Media analysis supplementary file

Inclusion & exclusion criteria:

| **Include** | **Exclude** |
| --- | --- |
| News outlets: ABC (Australian Government), Herald Sun (News Corp), Daily Telegraph (News Corp), Courier-Mail (News Corp), The West Australian (Seven West Media), The Australian (News Corp), Sydney Morning Herald (Nine Entertainment), The Age (Nine Entertainment), Canberra Times (Australian Community Media), Northern Territory News (News Corp), The Guardian (The Scott Trust), Hobart Mercury (News Corp). | - All other news outlets. |
| - 5-year timeframe from search date. | - Any articles published outside the 5 year timeframe. |
| - English language. | - Other languages. |
| - Articles focus on meat production or consumption and human health, or environmental health.   - Example: Livestock’s effects on climate change.   - Example: Vegetarian diets compared to omnivore diets and their effects on health/environment. - Articles focusing on solutions to address negative health or environmental impacts.   - Example: Feed additives to reduce cattle methane emissions.   - Example: Comparing plant-based alternatives to meat specifically. - Meat consumption or production and its association with human or environmental health must be a main topic in the article.   - Main topic: At least half of the article’s reporting focuses on either of the two.   - OR the title refers to them. | - Articles unrelated to meat and health/environment. - Articles where the main focus is not meat and health/environment. - Articles that cover a wide range of health/environmental issues and superficially mention meat.   - Example: Climate change generally, which also mentions meat’s impact. - Articles that have a different main topic which superficially relates to meat and health/environment.   - Example: A trade dispute with the EU because of deforestation practices in AUS which mentions livestock farming.   - Example: Animal diseases.   - Example: Celebrities and their overall diets, including vegetarian. - Anything related to fish or fish meat - Articles that focus on livestock more generally rather than meat specifically. - Articles that compare plant-based or vegan diets omnivore diets but do not specifically refer to meat. - Shorter versions of existing articles (duplicates in different news outlets which are only excerpts from included articles) |
| - News articles, commentary pieces, opinion pieces, analyses, blog articles. | - Any other type of news publication. |

Supplementary Table 1: Inclusion and exclusion criteria

| **Benefits of meat production/consumption** | **Risks of meat production/consumption** |
| --- | --- |
| Beneficial physical health outcomes (bone health, mortality, muscle mass, exercise strength, sexual health, cardiovascular health, cholesterol…) | Adverse physical health outcomes (cardiovascular disease, cancer, diabetes, weight gain, bone health, mortality, longevity, sexual health, cholesterol, antimicrobial resistance…) |
| Beneficial nutritional outcomes (protein, iron, zinc, magnesium, selenium, Vitamin Bs...) | Adverse nutritional outcomes (saturated fats, nutrient imbalance, lack of diversity in diet, foodborne illnesses, toxins, antibiotics…) |
| Beneficial mental health outcomes (daily energy, mental health conditions, mood, neurodevelopment…) | Adverse mental health outcomes (mood, mental health conditions, cognitive decline…) |
| Cultural or lifestyle benefits (social or traditional importance of meat consumption for well-being, meat as an evolutionary necessity, ‘we have always eaten meat’…) | Greenhouse gas emissions (methane, CO2, nitrogen, comparison with plant-based foods…) |
| Environmental management (Sustainable grazing systems, Carbon sequestration, Land use efficiency…) | Land use (land degradation, amount of land used for animal agriculture, deforestation, biodiversity loss due to land clearing…) |
| Technological improvements (reduced emissions, feed additives, methane-reduction, precision agriculture, circular economy approaches…) | Water use and pollution (water consumption, pollution through runoff from manure, pesticides, antibiotics, overextraction of local water resources…) |
| Ecosystem benefits (biodiversity conservation, wildlife management…) | Environmental contamination (antibiotics and hormones, fertilisers and pesticides, chemical pollution from meat processing…) |
| Economic benefits (rural livelihoods at stake, smallholder farmers survival, indigenous communities…) | Inefficiency of resource use (high input for low calorie/protein output, opportunity costs for land and energy use…) |
|  | General ethical and sustainability concerns (industrial animal agriculture’s impact on long-term sustainability, global comparisons with other countries, fundamental ethical issues of animal agriculture…) |

Supplementary Table 2: Benefits and risks of meat production/consumption

Supplementary Figure 1: PRISMA Flowchart

**Included**

Studies included in analysis **(n = 147)**

Studies screened **(n = 1500)**

Studies excluded **(n = 1353)**

**Screening**

**Identification**

References removed **(n = 112)**

Duplicates identified manually (n = 81)

Duplicates identified by Covidence (n = 31)

Studies from databases/registers **(n = 1612)**

Data extraction & coding:

- Title
- Newspaper name
- Author
- Date of publication
- Article type: News report, opinion piece, editorial …
- Primary topic: Health or environment or both
- Secondary topic: Meat consumption and health, vegan/vegetarian diet and health, Meat production and environment…
- Framing of meat headline: Favourable, slightly favourable, neutral, slightly unfavourable, unfavourable. Based on:
  - Tone of headline. Emphasis on benefit (health, environment) or risk (health, environment). Benefit = Favourable; Risk = Unfavourable. For more guidance, see next point.
- Framing of meat body of article: Favourable, slightly favourable, neutral, slightly unfavourable, unfavourable. Based on:
  - Does the body of the article focus mostly on benefits of meat or problems. Common benefits and problems for **health-related** articles:
  - Benefits:
    - Beneficial physical health outcomes (bone health, mortality, muscle mass, exercise strength, sexual health, cardiovascular health, cholesterol…)
    - Beneficial nutritional outcomes (protein, iron, zinc, magnesium, selenium, Vitamin Bs...)
    - Beneficial mental health outcomes (daily energy, mental health conditions, mood, neurodevelopment…)
    - Cultural or lifestyle benefits (social or traditional importance of meat consumption for well-being, meat as an evolutionary necessity, ‘we have always eaten meat’…)
  - Risks:
    - Adverse physical health outcomes (cardiovascular disease, cancer, diabetes, weight gain, bone health, mortality, longevity, sexual health, cholesterol, antimicrobial resistance…)
    - Adverse nutritional outcomes (saturated fats, nutrient imbalance, lack of diversity in diet, foodborne illnesses, toxins, antibiotics…)
    - Adverse mental health outcomes (mood, mental health conditions, cognitive decline…)
  - Does the body of the article focus mostly on benefits of meat or problems. Common benefits and problems for **environmentally related** articles:
  - Benefits:
    - Environmental management (Sustainable grazing systems, Carbon sequestration, Land use efficiency…)
    - Technological improvements (reduced emissions, feed additives, methane-reduction, precision agriculture, circular economy approaches…)
    - Ecosystem benefits (biodiversity conservation, wildlife management…)
    - Economic benefits (rural livelihoods at stake, smallholder farmers survival, indigenous communities…)
  - Risks:
    - Greenhouse gas emissions (methane, CO2, nitrogen, comparison with plant-based foods…)
    - Land use (land degradation, amount of land used for animal agriculture, deforestation, biodiversity loss due to land clearing…)
    - Water use and pollution (water consumption, pollution through runoff from manure, pesticides, antibiotics, overextraction of local water resources…)
    - Environmental contamination (antibiotics and hormones, fertilisers and pesticides, chemical pollution from meat processing…)
    - Inefficiency of resource use (high input for low calorie/protein output, opportunity costs for land and energy use…)
    - General ethical and sustainability concerns (industrial animal agriculture’s impact on long-term sustainability, global comparisons with other countries, fundamental ethical issues of animal agriculture…)
- Mention of scientific studies or referencing researchers: yes/no
- Call to action/solution: dietary change, policy change, business as usual, technological fix, individual responsibility, no call to action…
- Source classification:
  - Industry
    - Corporations/companies (JBS, Primo, Retailers like Coles…)
    - Industry associations (MLA, NFF…)
    - Individuals (Butchers, Farmers…)
  - Academia:
    - Independent
    - Industry affiliated
  - NGOs/Advocacy groups:
    - Environmental (Greenpeace, WWF…)
    - Health (PHAA, Cancer Council Australia…)
    - Animal Welfare (PETA…)
    - Farming (Farmers for Climate Action…)
  - Government institutions (Health department, environmental agencies, dietary guidelines…)
  - Politicians
  - Intergovernmental organisations (WHO, IPCC…)
  - Individuals from the public (Consumers, Influencers…)
  - Other: Text box
- For each source: Name of person or organisation, Number of times cited
- Article disclosures: COI, Funding…

Search string Factiva:

https://global-factiva-com.ap1.proxy.openathens.net/en/du/headlines.asp?napc=S&searchText=(meat*+OR+beef+OR+cattle+OR+pork+OR+chicken+OR+livestock+OR+%22animal+agriculture%22+OR+vegetarian+OR+%22animal+protein%22+OR+veggie+OR+plant-based+OR+vegan+OR+carnivore)+AND+(health*+OR+diet+OR+nutri*+OR+disease*+OR+cancer*+OR+overweight+OR+obesity+OR+diabetes+OR+cholesterol+OR+mortality+OR+unhealth*+OR+%22greenhouse+gas*%22+OR+emission*+OR+CO2+OR+climate+OR+environment*+OR+sustainab*+OR+biodiversity+OR+deforestation+OR+%22carbon+footprint%22+OR+pollution+OR+%22water+use%22+OR+%22land+use%22+OR+global+warming+OR+methane)+NOT+(corona*+OR+covid*)&exclude=Obituaries|Recurring&dateRangeMenu=date+after+-1835&sortBy=o&findIn=hlp&currentSources=U%7csmhh%2csmhhol%2cagee%2cageeol%2cnlhrsw%2chersun%2csfcour%2csfdate%2csfaust%2cnlhbmo%2cmrcury%2ctwau%2ccanbtz%2capngub%2cabcnew&currentSourcesDesc=sc_u_smhh%2cThe+Sydney+Morning+Herald%3bsc_u_smhhol%2cThe+Sydney+Morning+Herald+-+Online%3bsc_u_agee%2cThe+Age+(Melbourne%2c+Australia)%3bsc_u_ageeol%2cThe+Age+-+Online+(Melbourne%2c+Australia)%3bsc_u_nlhrsw%2cHerald+Sun+-+Online+(Melbourne%2c+Australia)%3bsc_u_hersun%2cHerald-Sun+(Melbourne%2c+Australia)%3bsc_u_sfcour%2cCourier+Mail+-+All+sources%3bsc_u_sfdate%2cDaily+Telegraph+(Australia)+-+All+sources%3bsc_u_sfaust%2cThe+Australian+-+All+sources%3bsc_u_nlhbmo%2cHobart+Mercury+-+Online+(Tasmania%2c+Australia)%3bsc_u_mrcury%2cHobart+Mercury+(Tasmania%2c+Australia)%3bsc_u_twau%2cThe+West+Australian+(Perth)%3bsc_u_canbtz%2cCanberra+Times+(ACT%2c+Australia)%3bsc_u_apngub%2cThe+Guardian+(Bundaberg%2c+Australia)%3bsc_u_abcnew%2cAustralian+Broadcasting+Corporation+News&searchLanguage=custom&searchLang=EN&dedupe=1&srchuiver=2&accountid=9UNI018500&namespace=18
